# Conventional Versus Virtual Reality-Based Hess–Lancaster Assessment: Agreement and Repeatability in Ocular Motility Evaluation

**DOI:** 10.3390/jemr19030067

**Published:** 2026-06-09

**Authors:** Francisco Javier Povedano-Montero, Álvaro Perales-Serrano, Daniela León Lobo, Rut González-Jiménez, Ricardo Bernárdez-Vilaboa, Juan E. Cedrún-Sánchez

**Affiliations:** 1 Optometry and Vision Department, Faculty of Optics and Optometry, Complutense University of Madrid, 28037 Madrid, Spainrutgon03@ucm.es (R.G.-J.);; 2 Hospital Doce de Octubre Research Institute (i+12), 28041 Madrid, Spain; 3 Applied Vision Research Group, Faculty of Optics and Optometry, Universidad Complutense de Madrid, 28037 Madrid, Spain

**Keywords:** Hess–Lancaster test, virtual reality, Dicopt Pro, ocular motility, strabismus, Bland–Altman analysis, repeatability

## Abstract

Background: The conventional Hess–Lancaster test is widely used to assess ocular misalignment across diagnostic gaze positions, but it relies on subjective responses and manual recording. Virtual reality may provide a more standardized framework for ocular motility assessment. Objectives: To evaluate the agreement and within-method repeatability of point-by-point deviation measurements obtained with the conventional Hess–Lancaster test and a VR-based Hess–Lancaster assessment implemented in Dicopt Pro. Methods: This cross-sectional observational study included 52 adults with suspected or diagnosed ocular motility disorders. Participants underwent both assessments using the same predefined gaze positions. Agreement was assessed using Bland–Altman analysis, concordance correlation coefficients, mean absolute differences, and mixed-effects modeling. Repeatability was evaluated in a subset with repeated measurements using session-to-session differences and intraclass correlation coefficients. Results: The VR-based assessment showed moderate agreement with the conventional test, with a mean concordance correlation coefficient of 0.57 for both eyes. Mean bias was 1.22 prism diopters for the right eye and 0.10 prism diopters for the left eye. Repeatability estimates were moderate-to-good, with ICC values ranging from 0.62 to 0.83, although repeated measurements were available only in a subset of participants. Conclusions: The VR-based Hess–Lancaster assessment showed small mean differences and moderate agreement with the conventional test, although both methods should be interpreted within the context of the complete clinical examination.

## 1. Introduction

Accurate quantification of ocular misalignment across different gaze positions is fundamental in the clinical evaluation of incomitant strabismus and paralytic ocular motility disorders [1,2]. Although prism cover testing provides an angular estimate of deviation in primary position, it does not characterize gaze-dependent changes that are often essential for diagnosing muscle paresis patterns and planning management [1]. For this reason, screen-based mapping techniques such as the Hess test have historically been used to represent binocular misalignment across the classical nine-position gaze framework, allowing systematic assessment of gaze-dependent ocular deviation patterns [2,3].

Since its original description in the early twentieth century, the Hess screen has remained a cornerstone in orthoptic and neuro-ophthalmological practice [2,4]. By dissociating binocular vision through color separation and plotting perceived alignment errors, the test enables inference of muscle underaction, secondary contracture, and incomitance patterns. However, the conventional procedure depends on subjective patient responses and manual plotting by the examiner, introducing potential sources of variability related to sensory adaptation, fixation instability, and graphical interpretation [3].

Methodological comparisons between dissociative screen tests have demonstrated that measurement outcomes may vary according to test geometry and viewing distance. In particular, divergence-related bias has been described when comparing the Hess and Harms screen techniques, suggesting that the recorded deviation magnitude can be influenced by the experimental configuration [5]. Such findings underscore that screen-based measurements are not purely intrinsic ocular values but are partially dependent on test design.

Efforts to increase objectivity in ocular motility assessment have evolved over several decades. Three-dimensional adaptations of the Hess paradigm employing scleral search coils demonstrated the feasibility of recording binocular eye rotations with high spatial precision, including torsional components [6]. These investigations provided valuable insight into ocular kinematics but remained limited to research settings due to their invasive nature and technical complexity. More clinically accessible approaches subsequently emerged, including head-mounted digital video systems capable of quantifying strabismus deviations in portable formats [7]. Additionally, the integration of eye-tracking into Hess or Lancaster-type paradigms has shown that subjective alignment tasks can be transformed into coordinate-based recordings, allowing direct capture of gaze position during structured motility testing [8].

Parallel to these developments, immersive virtual reality (VR) platforms have increasingly been applied in visual science, ophthalmology, and neuro-visual rehabilitation [9,10,11]. VR systems can provide standardized stimulus presentation, controlled viewing conditions, and digital recording of responses within a closed visual environment. When adapted to oculomotor assessment, these characteristics may improve procedural standardization and facilitate structured data storage. This aligns with the broader movement toward digital and data-supported clinical assessment in ophthalmology [12]. However, the degree of objectivity depends on the measurement strategy used. In VR-based Hess–Lancaster implementations that rely on participant alignment responses, the task remains subject-dependent, even if the data are recorded digitally.

Importantly, modernization of measurement techniques should preserve the diagnostic architecture that gives the Hess test its clinical value. The classical nine-position framework provides a symmetrical representation of gaze-dependent deviations and remains central to clinical interpretation of incomitant strabismus [2,3]. Several previous objective approaches modified task geometry or reduced positional sampling, thereby limiting direct comparability with conventional Hess charts [6]. Demonstrating agreement between a traditional Hess screen and an immersive coordinate-based method that maintains nine identical diagnostic positions therefore represents a necessary methodological step.

Beyond replication of the conventional chart, digital recording of Hess–Lancaster responses may provide structured coordinate-based outputs that can be stored, reviewed, and compared across visits. This may be useful for standardized documentation and follow-up, while reducing some limitations associated with manually plotted charts. Vector-based representation of deviations across gaze positions may also support a more systematic characterization of incomitance in future studies [13,14]. In the longer term, structured digital outputs could facilitate computational analysis of ocular motility patterns, as suggested by previous work on eye-movement classification and machine learning applied to eye-tracking data [15,16,17]. In the current protocol, however, no automated classification or eye-tracking-based measurement was performed.

However, despite these technological advances, evidence directly comparing conventional Hess–Lancaster measurements with VR-based implementations remains limited. In particular, formal agreement studies are needed to determine how closely these approaches correspond when the same diagnostic gaze positions and measurement scale are used. From a methodological perspective, validation of emerging digital assessment tools requires agreement and repeatability analyses against established clinical methods, while recognizing that statistical association alone does not demonstrate clinical interchangeability [18].

The present study compares measurements obtained with the conventional Hess–Lancaster test and those recorded using a VR-based implementation with digital recording capability (Dicopt Pro), preserving the same predefined diagnostic gaze positions in both modalities. Both systems were configured to use the same clinical measurement scale, with each grid unit corresponding to approximately 5 prism diopters, allowing direct comparison without post hoc conversion factors. However, the conventional test was performed on a physical screen at 60 cm, whereas the VR assessment used a simulated distance of 90 cm within a head-mounted display environment. Repeated measurements were also performed within each method, enabling evaluation of within-method repeatability.

The aim of this study was to evaluate the agreement between the conventional Hess–Lancaster test and a virtual reality-based Hess–Lancaster assessment using the same nine-position framework. A secondary objective was to assess within-method repeatability for both procedures. We hypothesized that the virtual reality-based assessment would show limited systematic bias and acceptable within-method repeatability, while recognizing that agreement may vary across gaze positions and may be influenced by testing geometry.

## 2. Materials and Methods

### 2.1. Study Design and Participants

A cross-sectional observational study was conducted between October 2025 and February 2026 at the Ophthalmology Department of Hospital Clínico San Carlos (Madrid, Spain). Participants were consecutively recruited from the Pediatric Ophthalmology and Strabismus Unit.

A total of 54 participants were initially enrolled. After applying exclusion criteria, 2 participants were excluded, resulting in a final sample of 52 participants included in the analysis.

Inclusion criteria comprised:

- age ≥ 18 years,
- clinical diagnosis or suspicion of oculomotor disorder (e.g., strabismus, cranial nerve palsy, or mechanical restriction)
- best-corrected visual acuity of at least 0.5 (decimal) in each eye, and
- sufficient cognitive ability to understand and perform the tests.

Exclusion criteria included severe ocular pathology interfering with fixation or binocular vision; ocular surgery within the previous 6 months; neurological conditions affecting ocular motility; and inability to tolerate or properly use the VR headset.

The study adhered to the tenets of the Declaration of Helsinki and was approved by the Institutional Ethics Committee of Hospital Clínico San Carlos (approval code: 25/147-E, 6 March 2025). Written informed consent was obtained from all participants.

**Figure 1:**
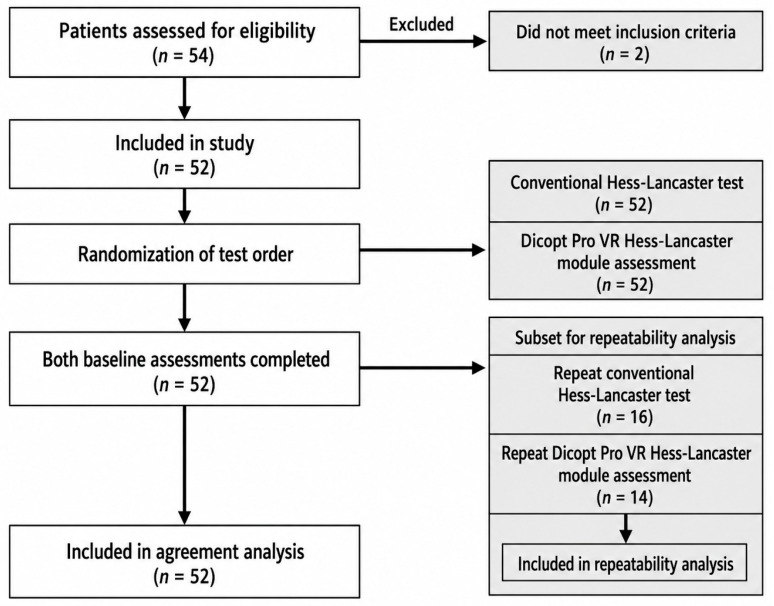
Study flow diagram.

The study workflow, including participant selection, randomization, and assessment procedures, is summarized in Figure 1.

### 2.2. Sample Size Considerations

Sample size estimation was based on the comparison of paired measurements between two methods. A minimum clinically relevant difference equivalent to one Hess–Lancaster grid unit was considered, and the expected variability of paired differences was expressed on the same grid-unit scale. Under these assumptions, with a 95% confidence level and 80% statistical power, the minimum required sample size was estimated at 36 participants. The final sample of 52 participants exceeded this requirement, providing adequate precision for the primary agreement analysis.

### 2.3. Instrumentation

Conventional Hess–Lancaster Test

The traditional Hess–Lancaster screen consisted of a red grid (108 cm × 108 cm) with 36 × 36 squares (3 cm each). The assessment was performed at a viewing distance of 60 cm, resulting in each grid unit corresponding to approximately 5 prism diopters (Δ). Binocular dissociation was achieved using red–green filters, and measurements were obtained using a red laser pointer stimulus, allowing evaluation of ocular deviation across the Hess–Lancaster gaze positions.

Virtual Reality System (Dicopt Pro)

The virtual assessment was performed using the Pico Neo 3 Pro Eye headset (Pico Interactive Inc., Beijing, China) together with Dicopt Pro software version 1.7.1 (Innotec Vision Avanzada XR, SL, Madrid, Spain). Dicopt Pro includes a virtual reality-based Hess–Lancaster module that reproduces the test in an immersive environment using dichoptic stimulus presentation.

The virtual test was configured at a simulated distance of 90 cm, with each grid unit corresponding to approximately 5 prism diopters. Stimuli were presented sequentially across the diagnostic gaze positions, and responses were recorded automatically in digital format.

Although the device incorporates eye-tracking technology, it was not used for measurement acquisition in this protocol, and responses remained subject-dependent.

**Figure 2:**
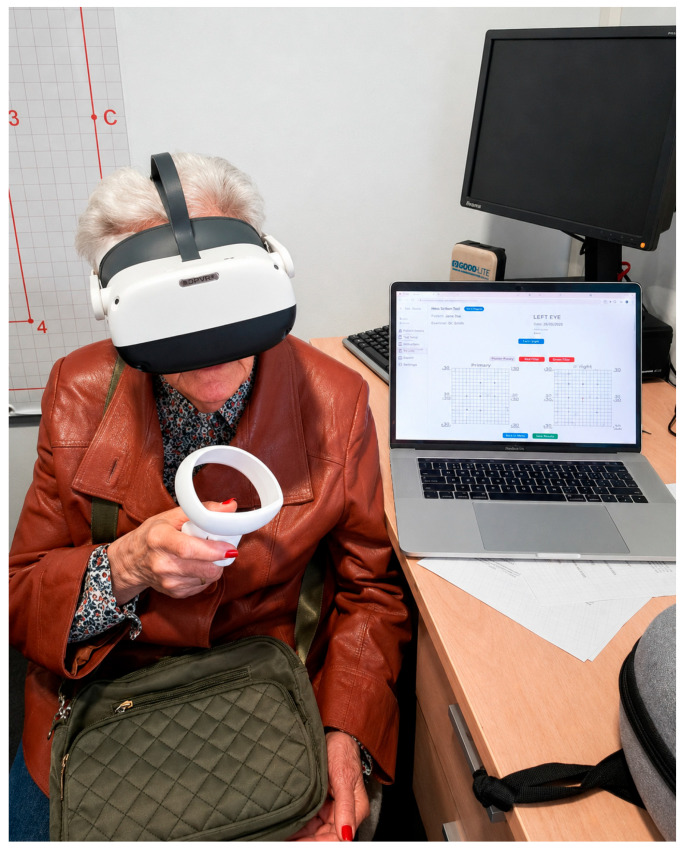
Virtual reality-based Hess–Lancaster assessment using Dicopt Pro.

The virtual reality-based Hess–Lancaster assessment setup is shown in Figure 2. During the test, participants wore the head-mounted display and used a handheld controller to align the virtual pointer with the sequentially presented stimuli. The examiner monitored the procedure through the connected computer interface, where the coordinate-based responses were automatically recorded in digital format.

### 2.4. Scale Standardization Between Methods

Both systems were configured to use the same clinical measurement scale, with each grid unit corresponding to approximately 5 prism diopters in both modalities. This allowed deviations recorded with the conventional Hess–Lancaster test and Dicopt Pro to be compared without post hoc conversion factors. However, this scale standardization should not be interpreted as full equivalence of the two testing environments, since the conventional test was performed at a physical distance of 60 cm, whereas the VR assessment used a simulated distance of 90 cm.

A detailed comparison of the main methodological characteristics of both assessment procedures is provided in Supplementary Table S1.

### 2.5. Experimental Procedure

All participants underwent both assessment methods, the conventional Hess–Lancaster test and the Dicopt Pro virtual reality assessment, within a single session. Agreement analysis between methods was performed using the full sample of 52 participants. Within-method repeatability was assessed in a subset of participants who completed repeated measurements during the same session. Repeated conventional Hess–Lancaster measurements were available for 16 participants, whereas repeated Dicopt Pro measurements were available for 14 participants after excluding incomplete repeated recordings. This subset was used specifically to evaluate session-to-session consistency within each method.

To minimize potential order effects, including learning, fatigue, or adaptation, the sequence of the two assessment methods (conventional Hess–Lancaster and virtual reality test) was randomized across participants. This randomization ensured that neither method systematically benefited from being performed first.

While the order of the two methods was randomized, the sequence of stimulus presentation within each test followed a predefined standardized order.

For the conventional test, participants were positioned at 60 cm from the screen with head stabilization (chin and forehead support). After binocular dissociation using red–green filters, the examiner projected a reference stimulus while the participant aligned a luminous target across the nine diagnostic gaze positions. Measurements were recorded manually on a standardized grid.

Point stimuli were used to assess ocular deviation across the nine diagnostic gaze positions. The procedure was repeated after reversing the filters to evaluate both eyes.

For the virtual reality test, dichoptic stimulus presentation was used, whereby each eye received different visual information simultaneously to achieve binocular dissociation within the immersive environment. Participants used a handheld controller to align a virtual pointer with the sequentially presented stimuli. Each position was confirmed by maintaining alignment and pressing the trigger button. The system automatically recorded deviations in prism diopters and gaze coordinates.

Stimuli were presented in a predefined sequence covering the nine diagnostic gaze positions, ensuring consistency across participants and between repeated assessments.

### 2.6. Data Structure and Variables

The primary outcome variable was the point-by-point deviation measured in prism diopters at each of the nine diagnostic gaze positions for both assessment methods.

Each gaze position was analyzed as an individual observation. Therefore, each participant contributed multiple data points across gaze positions, methods (conventional Hess–Lancaster vs. Dicopt Pro), repetitions, and eyes, reflecting the repeated-measures structure of the dataset.

As a result, the dataset had a hierarchical structure, with measurements nested within gaze positions, repetitions, methods, eyes, and ultimately within participants. Gaze positions were analyzed as separate point-coordinate observations in order to preserve the structure of the Hess–Lancaster assessment. However, these observations were not statistically independent. For this reason, agreement results were interpreted in light of the repeated-measures structure, and mixed-effects models were used as complementary analyses to account for within-subject clustering.

This approach allowed evaluation of:

- agreement between methods at the level of individual gaze positions,
- within-method repeatability, and
- the influence of gaze position on measurement variability.

For the main agreement analysis, no aggregation of measurements was performed prior to analysis, preserving the full point-coordinate data structure. A participant-eye aggregated analysis was subsequently performed as a sensitivity analysis.

### 2.7. Statistical Analysis

Statistical analysis was performed using SPSS 30 (IBM Corp., Armonk, NY, USA) and R version 4.5.3 (R Foundation for Statistical Computing, Vienna, Austria). Statistical significance was set at *p* < 0.05.

Continuous variables were summarized as mean ± standard deviation (SD). The normality of the differences between methods was assessed using the Shapiro–Wilk test.

To account for the hierarchical structure of the dataset and repeated measurements within participants, method and gaze position were included as fixed effects, and participant was included as a random effect.

A complementary analytical approach was used because each statistical method addressed a different aspect of the comparison between tests. Bland–Altman analysis was used to estimate mean bias and limits of agreement between methods, whereas concordance correlation coefficients were used to summarize overall agreement by combining precision and accuracy. Intraclass correlation coefficients were used to assess within-method repeatability, and mixed-effects models were applied to evaluate systematic method-related differences while accounting for the repeated-measures structure of the dataset. Therefore, *p*-values from paired comparisons or mixed-effects models were interpreted as evidence of systematic differences, not as evidence of clinical agreement or interchangeability.

#### 2.7.1. Agreement Analysis

Agreement between the conventional Hess–Lancaster test and the VR-based Hess–Lancaster assessment performed with Dicopt Pro was primarily evaluated using Bland–Altman analysis and concordance correlation coefficients (CCC). Bland–Altman analysis was used to describe the average difference between methods and the expected range of individual differences, whereas CCC values were used as a complementary summary of global concordance, combining measures of precision and accuracy.

Mean differences (bias) and 95% limits of agreement (LoA; mean difference ± 1.96 SD) were calculated.

As multiple observations per participant were included, Bland–Altman plots were constructed using all paired measurements across gaze positions. Agreement analyses were conducted at the measurement level using paired point-coordinate observations and were interpreted considering the repeated-measures structure of the dataset. To examine whether the main findings were influenced by within-subject clustering, a sensitivity analysis was also performed after aggregating point-coordinate measurements at the participant-eye level. Regression analysis of the differences against the mean values was performed to assess proportional bias.

#### 2.7.2. Repeatability Analysis

Within-method repeatability was assessed separately for each method using intraclass correlation coefficients (ICC). A two-way mixed-effects model with absolute agreement for single measurements was applied. The 95% confidence intervals for ICC values were also calculated. ICCs were calculated separately for each method and eye using paired repeated point-coordinate measurements obtained during the same session. Repeatability was also assessed by comparing the two repeated measurements within each method at each gaze position.

As additional clinically interpretable repeatability metrics, the within-subject standard deviation (Sw) and coefficient of repeatability (CoR) were calculated from the differences between repeated measurements. Sw was estimated as the standard deviation of the session-to-session differences divided by √2, and CoR was calculated as 1.96 times the standard deviation of the differences. These indices were calculated using participant-eye aggregated repeated measurements to reduce the influence of non-independent point-coordinate observations.

#### 2.7.3. Comparison Between Methods

Paired comparisons between methods were performed using paired *t*-tests for normally distributed data or Wilcoxon signed-rank tests otherwise. These analyses were considered complementary to agreement analysis, as statistical significance does not necessarily reflect clinical agreement.

#### 2.7.4. Mixed-Effects Modeling

To account for the hierarchical structure of the data and repeated measurements within participants, linear mixed-effects models were applied using deviation values as the dependent variable. Method, eye, gaze position/component, and the method-by-eye interaction were included as fixed effects, while participant was included as a random effect. This model was used as a complementary analysis to evaluate systematic method-related differences while accounting for within-subject clustering.

#### 2.7.5. Handling of Measurement Scale

As both systems were standardized to the same measurement scale (5 prism diopters per grid unit), no data transformation or conversion factors were required prior to analysis.

## 3. Results

### 3.1. Sample Characteristics

A total of 52 participants were included in the final analysis. The mean age was 58.0 ± 20.0 years. The sample included 33 female and 19 male participants.

### 3.2. Agreement Between Conventional Hess–Lancaster and VR-Based Hess–Lancaster Assessment

**Figure 3:**
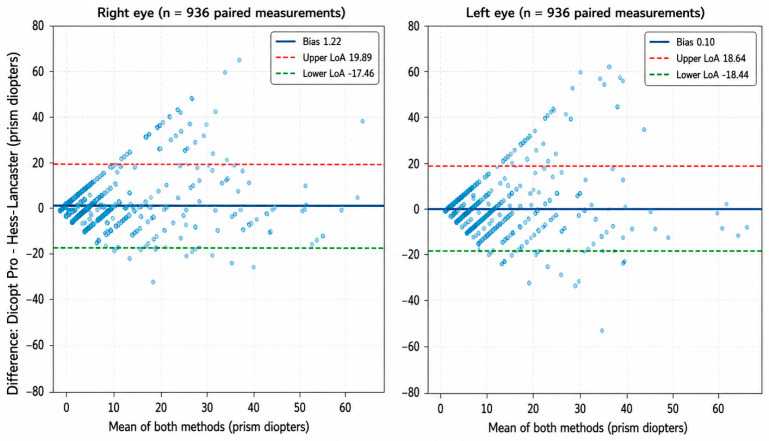
Bland–Altman plots showing point-by-point agreement between the conventional Hess–Lancaster test and the VR-based Hess–Lancaster assessment performed with Dicopt Pro for the right and left eyes. Blue circles represent individual paired point-coordinate measurements. The solid blue line indicates the mean difference, and the dashed red and green lines indicate the upper and lower 95% limits of agreement, respectively.

Bland–Altman analysis was performed to evaluate agreement between the conventional Hess–Lancaster test and the VR-based Hess–Lancaster assessment performed with Dicopt Pro using deviation measurements (Figure 3). For each eye, the analysis included 936 paired point-coordinate measurements, corresponding to 52 participants assessed across nine diagnostic gaze positions and two coordinate components. Global agreement results are summarized in Table 1.

For the right eye, the mean bias was 1.22 prism diopters, with 95% limits of agreement ranging from −17.46 to 19.89 prism diopters. For the left eye, the mean bias was 0.10 prism diopters, with 95% limits of agreement ranging from −18.44 to 18.64 prism diopters. The mean CCC was 0.57 for both eyes, and the mean absolute differences were 4.78 and 4.59 prism diopters for the right and left eyes, respectively. Overall, these findings indicate limited systematic bias, although some variability was observed at the individual measurement level. The complete point-by-point agreement analysis by eye, diagnostic gaze position, and coordinate component is provided in Supplementary Table S2.

As a sensitivity analysis, agreement was also evaluated after aggregating point-coordinate measurements at the participant-eye level (Supplementary Table S3). This analysis showed similar mean differences between methods, with narrower limits of agreement and higher CCC values, supporting the robustness of the main findings while reducing the influence of within-subject clustering.

### 3.3. Agreement According to Diagnostic Gaze Position

**Figure 4:**
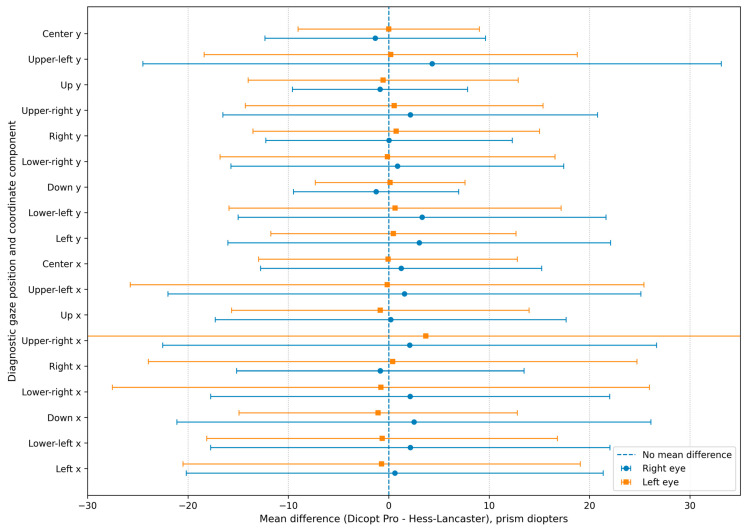
Forest plot of point-by-point agreement between the conventional Hess–Lancaster test and VR-based Hess–Lancaster assessment performed with Dicopt Pro.

Figure 4 shows the point-by-point agreement between the conventional Hess–Lancaster test and Dicopt Pro according to diagnostic gaze position and coordinate component. Overall, mean differences between methods were generally close to zero in most gaze positions, suggesting limited systematic bias across the visual field. However, the width of the limits of agreement varied depending on the gaze position, indicating that agreement was not completely uniform across all diagnostic positions.

Greater variability was observed in some oblique and horizontal gaze components, whereas several vertical and central components showed narrower limits of agreement. This pattern suggests that the agreement between both methods may depend partly on the gaze direction assessed, with some positions showing more stable correspondence than others.

Agreement according to diagnostic gaze position is summarized in Table 2 and illustrated in Figure 4. Table 2 presents the positions showing the highest and lowest agreement, while the full point-by-point results are reported in Supplementary Table S2. The highest agreement was observed for the lower gaze y-axis component, followed by the upper and central gaze components. In contrast, lower CCC values were observed in some oblique and horizontal components, particularly in the upper-left and lower-left gaze positions.

### 3.4. Within-Method Repeatability

**Figure 5:**
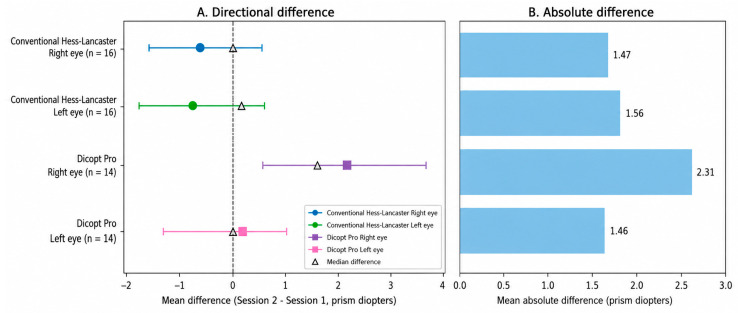
Within-method repeatability analysis for paired measurements.

Within-method repeatability was assessed in the subset of participants with repeated measurements available. Repeated conventional Hess–Lancaster measurements were available for 16 participants, whereas repeated Dicopt Pro measurements were available for 14 participants. Repeatability analysis is shown in Figure 5 and summarized in Table 3 and Table 4. ICC values were calculated using paired repeated point-coordinate measurements, whereas mean session-to-session differences and mean absolute differences were summarized at the participant-eye level. The complete repeatability analysis by method, eye, diagnostic gaze position, and coordinate component is provided in Supplementary Table S4.

The conventional Hess–Lancaster test showed small directional differences between the first and second measurements in both eyes. Dicopt Pro also showed relatively small between-session differences overall, although repeatability was less uniform, with lower repeatability for the right eye. ICC values suggested moderate-to-good within-method repeatability; however, these estimates should be interpreted cautiously because repeated measurements were available only for a subset of participants. Additional repeatability indices, including within-subject standard deviation and coefficient of repeatability, are reported in Table 4.

### 3.5. Mixed-Effects Model Analysis

Linear mixed-effects modeling was performed as a complementary analysis to evaluate systematic differences between methods while accounting for the repeated-measures structure of the dataset. Method, eye, gaze position/component, and the method-by-eye interaction were included as fixed effects, with participant as a random effect. The results of the model are summarized in Table 5.

The model showed a small but statistically significant method effect for the right eye, with Dicopt Pro measurements being on average 1.22 prism diopters higher than conventional Hess–Lancaster measurements (β = 1.22 Δ; 95% CI: 0.52 to 1.91; *p* = 0.001). The method-by-eye interaction was also significant (β = −1.11 Δ; 95% CI: −2.10 to −0.13; *p* = 0.026), indicating that the method-related difference was smaller for the left eye. Accordingly, the estimated method difference for the left eye was close to zero and not statistically significant (β = 0.10 Δ; 95% CI: −0.59 to 0.80; *p* = 0.772). Overall, these findings support the Bland–Altman results, showing limited average systematic bias but eye-dependent differences between methods.

## 4. Discussion

The present study evaluated the agreement and within-method repeatability of deviation measurements obtained with the conventional Hess–Lancaster test and the VR-based Hess–Lancaster assessment performed with Dicopt Pro. Overall, the VR-based assessment showed moderate agreement with the conventional test across the nine-position framework. Mean differences between methods were small, particularly for the left eye, but the limits of agreement indicated relevant point-by-point variability. For this reason, individual measurements should be interpreted together with the clinical findings and the gaze position assessed.

The global concordance correlation coefficient was 0.57 for both eyes, indicating moderate agreement between methods. This finding should be interpreted considering the characteristics of the conventional Hess–Lancaster test itself. Although the test remains clinically useful for mapping incomitant ocular deviations, it is not a fully objective reference standard. Patient responses, examiner interaction, manual plotting, binocular dissociation, fixation stability, and test geometry may all influence the measurements. Thus, differences between the conventional and virtual methods may reflect both factors related to the VR system and the intrinsic variability of the traditional procedure.

The complementary mixed-effects model supported these findings by showing limited average systematic bias after accounting for within-subject clustering, although the method-related difference was slightly greater for the right eye than for the left eye.

Automated, video-based, eye-tracking, augmented reality, and virtual reality approaches have been proposed to reduce examiner-dependent variability and improve standardization in ocular alignment assessment. Previous studies have shown that head-mounted video systems, eye-tracking-enhanced Hess–Lancaster paradigms, automated strabismus devices, and immersive VR implementations can provide quantitative measurements of ocular deviation, although they also emphasize the importance of measurement variability, calibration, and repeatability when translating these technologies into clinical practice [7,8,19,20,21]. The present study extends this line of work by directly comparing a VR-based Hess–Lancaster assessment with the conventional clinical test while preserving the same nine-position diagnostic framework and clinical measurement scale.

The Bland–Altman analysis showed a limited average systematic bias, suggesting that the VR-based assessment did not consistently overestimate or underestimate point-by-point deviation values. However, the relatively wide limits of agreement indicate that individual differences between methods may still be clinically relevant in some cases. These differences should be interpreted according to the gaze position, deviation magnitude, diagnosis, and intended use of the measurement, in line with methodological recommendations for agreement and repeatability studies [18,22].

Since each grid unit corresponds to approximately 5 prism diopters in both systems, differences below one grid unit may be interpreted cautiously as being within the practical resolution of the test. However, a single universal threshold for clinical relevance cannot be assumed. Previous VR-based validation work also supports considering device-specific scaling, systematic offsets, and testing conditions when interpreting quantitative outputs [23].

The width of the limits of agreement is clinically relevant. Although the mean bias between methods was small, individual point-by-point differences may be important in cases where high precision is required. Therefore, the VR-based Hess–Lancaster assessment should not be used as a standalone replacement for the conventional test in preoperative planning for extraocular muscle surgery or when precise surgical dosing is required. In these situations, Dicopt Pro results should be interpreted together with the complete orthoptic and ophthalmological examination, including prism cover testing, ocular motility assessment, binocular sensory evaluation, and other clinical criteria when appropriate. Thus, the present results support the use of the VR-based assessment as a complementary tool for digital documentation and follow-up, but not as evidence of complete clinical interchangeability between both methods.

Agreement was not uniform across diagnostic gaze positions, with greater variability in some oblique and horizontal components. This position-dependent variability is clinically relevant because incomitant deviations are inherently gaze-dependent.

Although both systems were standardized to the same prism-diopter scale, the conventional test was performed at a physical distance of 60 cm with head stabilization, whereas the virtual assessment was performed at a simulated distance of 90 cm within a head-mounted display environment. This difference may have modified the proximal convergence demand and contributed to variability in eccentric or oblique gaze positions, where spatial localization, vergence demand, headset alignment, and perceived target position may be more sensitive to test geometry. Previous comparisons between dissociative screen tests also support the influence of viewing distance and experimental configuration on recorded deviation values [5].

The present findings also align with previous VR-based studies of ocular misalignment. Nesaratnam et al. reported that a virtual reality-based test of ocular misalignment was feasible when compared with the traditional Lees screen but also emphasized the need for further validation before clinical implementation [24]. More recently, Wang et al. evaluated a virtual reality system for automated strabismus measurement and diagnosis using Bland–Altman plots and ICC analysis, showing the potential of VR-based methods but also reporting systematic differences between VR and manual measurements [25]. These studies support the idea that VR technology can provide a controlled and scalable environment for ocular alignment assessment, while also highlighting that device-specific calibration, testing distance, response modality, and patient-related factors remain important sources of variability.

Within-method repeatability was moderate to good for both approaches according to ICC values [26], although these estimates should be interpreted cautiously because repeated measurements were available only for a subset of participants. The additional within-subject standard deviation and coefficient of repeatability values provide a more clinically interpretable estimate of test–retest variability. In both methods, the coefficients of repeatability were close to one Hess–Lancaster grid unit, suggesting that part of the observed session-to-session variation may fall within the practical resolution of the test.

However, Dicopt Pro showed lower repeatability for the right eye, consistent with the larger session-to-session difference observed in that condition. This eye-dependent variability may reflect learning or adaptation to the virtual environment, controller response variability, fixation instability, clinical heterogeneity, headset alignment, spatial calibration, or the limited size of the repeatability subsample.

Because the order of gaze-position presentation within each test followed a predefined sequence, sequential learning, adaptation, or fatigue effects cannot be completely excluded. Manual controller use may also have introduced response variability, particularly during repeated VR measurements. For this reason, the repeatability findings should be considered preliminary and should not be interpreted as definitive evidence of software-related instability. Larger repeated-measures studies will be needed to confirm these results and to determine whether repeatability differs according to eye, gaze position, or clinical profile.

An important strength of this study is that both methods were compared using the same classical diagnostic gaze positions, preserving the clinical structure of the Hess–Lancaster test. This is relevant because several digital or automated approaches focus mainly on primary position or simplified gaze conditions. By maintaining the nine-position framework, the present study provides a more clinically meaningful comparison for incomitant deviations and paralytic or restrictive motility disorders. In addition, the use of Bland–Altman analysis, CCC, limits of agreement, and ICC provides a comprehensive methodological approach to evaluate agreement and repeatability.

**Figure 6:**
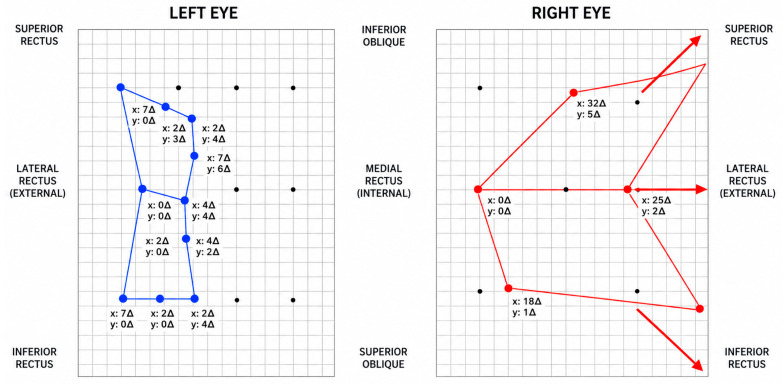
Representative VR-based Hess–Lancaster pattern in a patient with complex incomitant ocular deviation secondary to previous sinus surgery. The figure illustrates gaze-dependent asymmetry across the diagnostic positions and is presented as a descriptive clinical example.

Figure 6 shows a representative case of complex incomitant ocular deviation secondary to previous sinus surgery, assessed with the VR-based Hess–Lancaster module of Dicopt Pro. The digital grid shows a marked gaze-dependent asymmetry in ocular alignment, with a distorted pattern across the diagnostic positions. This example is included to illustrate the type of coordinate-based output generated by the VR system and its potential value for documenting incomitant patterns. However, it should be considered only as a descriptive clinical example, not as evidence of diagnosis-specific diagnostic accuracy.

The study also has limitations. First, the conventional Hess–Lancaster test was used as the clinical reference, although it is itself subject to variability and should not be considered a fully objective gold standard. Second, the repeatability analysis was performed in a smaller subset of participants, which limits the precision of the repeatability estimates and supports a cautious interpretation of the ICC, within-subject SD, and coefficient of repeatability values. Third, because multiple point-coordinate measurements were obtained from each participant, agreement estimates were interpreted at the measurement level and in the context of the repeated-measures structure of the dataset. Mixed-effects models and participant-eye aggregated sensitivity analyses were included as complementary approaches to reduce the influence of within-subject clustering.

One author had a commercial affiliation with Dicopt Pro; however, data interpretation and statistical analyses were performed collaboratively by the research team following predefined methodological criteria. Further independent studies in other clinical settings would be useful to confirm the reproducibility of these findings.

Fourth, although the headset incorporates eye-tracking technology, eye tracking was not used for measurement acquisition in this protocol, and responses remained subject-dependent. Therefore, the present results reflect the performance of a VR-based Hess–Lancaster assessment with digital recording, rather than a fully automated eye-tracking measurement system.

Fifth, although the sample included patients with suspected or diagnosed ocular motility disorders, it was not possible to stratify the analysis reliably by diagnosis, deviation type, or severity because some clinical subgroups were small and heterogeneous. This is an important limitation, especially for the Hess–Lancaster test, whose clinical value depends largely on the qualitative interpretation of incomitant patterns in conditions such as extraocular muscle palsy, restrictive motility disorders, or complex post-surgical deviations. For this reason, the pooled agreement analysis may not fully reflect the performance of the VR-based system in specific diagnostic groups. Future studies should compare conventional and VR-based Hess–Lancaster outputs in larger and better-defined subgroups, including comitant strabismus, cranial nerve palsies, restrictive disorders, post-surgical deviations, and small versus large deviations.

Future research should explore whether automated eye-tracking outputs can be integrated into VR-based Hess–Lancaster assessment and whether calibration procedures can be optimized for eccentric gaze positions. Recent evidence on automated and telemedicine-based strabismus assessment highlights the potential of digital tools to improve accessibility, reduce subjectivity, and support standardized documentation, while also emphasizing the need for robust validation against in-person clinical examination before widespread implementation [27,28]. In this context, the structured digital output generated by VR-based assessments may support future computational analyses of ocular motility patterns, but further technical refinement, external validation, and clinically stratified studies remain necessary.

## 5. Conclusions

The virtual reality–based Hess–Lancaster assessment performed with Dicopt Pro showed small mean differences and moderate agreement with the conventional Hess–Lancaster test across diagnostic gaze positions. However, the relatively wide limits of agreement indicate that individual point-by-point measurements should be interpreted within the context of the complete clinical examination rather than as directly interchangeable values. At present, the VR-based assessment should be considered a complementary digital tool rather than a standalone replacement for the conventional test, particularly in clinical decisions requiring high precision, such as preoperative planning for extraocular muscle surgery. Within-session repeatability estimates were moderate to good for both approaches, although these findings should be interpreted cautiously because repeated measurements were available only for a subset of participants. Overall, VR-based Hess–Lancaster assessment may provide a useful approach for standardized ocular motility documentation and follow-up, while further studies are needed to confirm its clinical performance in larger and diagnostically stratified samples.

## Figures and Tables

**Table 1:** Global agreement between the conventional Hess–Lancaster test and the VR-based Hess–Lancaster assessment performed with Dicopt Pro using individual measurements.

Eye	Mean CCC	Mean Difference (bias), Δ	95% LoA, Δ	Median Difference, Δ	Mean Absolute Difference, Δ
Right eye	0.57	1.22	−17.46 to 19.89	0.00	4.78
Left eye	0.57	0.10	−18.44 to 18.64	−0.06	4.59

**Table 2:** Selected diagnostic gaze positions showing higher and lower agreement between methods.

Gaze Position/Component	Mean CCC	Mean Difference, Δ	Median Difference, Δ	Mean Absolute Difference, Δ
Lower gaze, y-axis	0.84	−0.56	0.00	2.06
Upper gaze, x-axis	0.73	−0.32	0.00	4.36
Upper gaze, y-axis	0.72	−0.72	0.00	2.90
Central gaze, y-axis	0.70	−0.67	0.00	3.09
Right gaze, y-axis	0.70	0.38	0.00	3.06
Upper-left gaze, x-axis	0.44	0.70	−0.50	7.06
Left gaze, x-axis	0.43	−0.05	0.00	5.64
Upper-left gaze, y-axis	0.41	2.26	0.00	6.40
Lower-left gaze, y-axis	0.36	1.98	0.00	4.18

**Table 3:** Within-method repeatability based on participant-eye mean point-coordinate deviation measurements.

Method	Eye	*n*	Mean S1, Δ	Mean S2, Δ	Mean Difference S2–S1, Δ	Mean Absolute Difference, Δ	*p*-Value	ICC (A,1)	95% CI ICC
Conventional Hess–Lancaster	Right eye	16	4.60	4.21	−0.39	1.47	0.6948	0.73	0.61–0.82
Conventional Hess–Lancaster	Left eye	16	4.58	3.98	−0.61	1.56	1.0000	0.71	0.59–0.81
VR-based Hess–Lancaster assessment (Dicopt Pro)	Right eye	14	4.90	6.92	2.02	2.31	0.0159	0.62	0.51–0.74
VR-based Hess–Lancaster assessment (Dicopt Pro)	Left eye	14	6.15	6.31	0.17	1.46	0.5751	0.83	0.74–0.91

**Table 4:** Additional repeatability indices based on participant-eye aggregated repeated measurements.

Method	Eye	*n*	Mean Difference S2–S1, Δ	Mean Absolute Difference, Δ	Within-Subject SD, Δ	Coefficient of Repeatability, Δ
Conventional Hess–Lancaster	Right eye	16	−0.39	1.47	1.52	4.20
Conventional Hess–Lancaster	Left eye	16	−0.61	1.56	1.82	5.06
VR-based Hess–Lancaster assessment (Dicopt Pro)	Right eye	14	2.02	2.31	1.90	5.25
VR-based Hess–Lancaster assessment (Dicopt Pro)	Left eye	14	0.17	1.46	1.98	5.48

**Note:** ICC values correspond to a two-way mixed-effects model with absolute agreement for single measurements. The 95% confidence intervals were estimated using bootstrap resampling. Within-subject SD: within-subject standard deviation; coefficient of repeatability: 1.96 × SD of the session-to-session differences.

**Table 5:** Linear mixed-effects model evaluating systematic method-related differences.

Effect	Estimate, Δ	95% CI	*p*-Value	Interpretation
Method effect, right eye	1.22	0.52 to 1.91	0.001	Dicopt Pro showed slightly higher values than conventional Hess–Lancaster
Method × left eye interaction	−1.11	−2.10 to −0.13	0.026	The method-related difference was smaller for the left eye
Estimated method effect, left eye	0.10	−0.59 to 0.80	0.772	No significant systematic difference between methods

## Data Availability

The data presented in this study are available on reasonable request from the corresponding author. The data are not publicly available due to privacy and ethical restrictions, as they contain clinical information from human participants.

## Supplementary Materials

The following supporting information can be downloaded at: https://www.mdpi.com/article/10.3390/jemr19030067/s1, Table S1: Main methodological characteristics of the conventional Hess–Lancaster test and the VR-based Hess–Lancaster assessment; Table S2: Full point-by-point agreement analysis between the conventional Hess–Lancaster test and Dicopt Pro according to diagnostic gaze position and coordinate component; Table S3: Sensitivity analysis based on participant-eye aggregated measurements; Table S4: Full within-method repeatability analysis for point-by-point deviation measurements according to diagnostic gaze position and coordinate component.

## Abbreviations

The following abbreviations are used in this manuscript:

AI	Artificial intelligence
BA	Bland–Altman
CCC	Concordance correlation coefficient
CI	Confidence interval
ICC	Intraclass correlation coefficient
LoA	Limits of agreement
ML	Machine learning
SD	Standard deviation
S1	First session
S2	Second session
VR	Virtual reality
Δ	Prism diopters
CoR	Coefficient of repeatability
Sw	Within-subject standard deviation
