# Supplementary material for: Conventional Versus Virtual Reality-Based Hess–Lancaster Assessment: Agreement and Repeatability in Ocular Motility Evaluation
Source: J Eye Mov Res. 2026 Jun 9;19(3):67. doi: 10.3390/jemr19030067 (PMC13302422; doi:10.3390/jemr19030067)
Supplement: Supplementary file 1 [file jemr-19-00067-s001.zip › jemr-4358635-supplementary.pdf]

Supplementary Material

Conventional Versus Virtual Reality-Based Hess-Lancaster Assessment: Agreement and Repeatability in Ocular Motility Evaluation

Note. Only point-stimulus measurements are included, in line with the main analysis. Point-coordinate measurements should be interpreted considering the repeated-measures structure of the dataset, as multiple observations were obtained from each participant. Abbreviations: CCC, concordance correlation coefficient; LoA, limits of agreement; Δ, prism diopters; S1, first session; S2, second session.

Supplementary Table S1. Main methodological characteristics of the conventional Hess-Lancaster test and the VR-based Hess-Lancaster assessment.

Characteristic	Conventional Hess-Lancaster test	VR-based Hess-Lancaster assessment, Dicopt Pro
Testing environment	Physical Hess-Lancaster screen	Head-mounted virtual reality environment
Device / system	Red grid screen, 108 × 108 cm, with 3 cm grid squares	Pico Neo 3 Pro Eye headset with Dicopt Pro software
Viewing distance	Physical distance of 60 cm	Simulated viewing distance of 90 cm
Measurement scale	Approximately 5 prism diopters per grid unit	Approximately 5 prism diopters per grid unit
Diagnostic gaze positions	Classical nine-position Hess-Lancaster framework	Same predefined nine diagnostic gaze positions
Binocular dissociation	Red–green filters	Dichoptic stimulus presentation within the headset
Stimulus / alignment task	Examiner presents a luminous reference stimulus; participant aligns the target	Sequentially presented virtual stimuli; participant aligns a virtual pointer
Response mode	Manual alignment using conventional test procedure	Handheld controller; confirmation by trigger press
Recording method	Manual recording on standardized grid	Automatic digital recording of prism-diopter deviations and gaze coordinates
Output format	Conventional plotted Hess-Lancaster chart	Coordinate-based digital output
Head stabilization	Chin and forehead support	Head-mounted display; headset-based positioning
Eye tracking in current protocol	Not applicable	Available in the headset but not used for measurement acquisition
Nature of measurement	Subject-dependent alignment response	Subject-dependent alignment response with digital recording
Main practical advantage	Established clinical interpretation and familiar chart format	Standardized stimulus presentation, digital storage, and follow-up comparison

Supplementary Table S2. Full point-by-point agreement analysis between the conventional Hess-Lancaster test and Dicopt Pro according to diagnostic gaze position and coordinate component.

Eye	Position/component	n paired measurements	Mean difference, Δ	Median difference, Δ	Mean absolute difference, Δ	Lower LoA, Δ	Upper LoA, Δ	CCC
Left eye	Central gaze, x-axis	52	-0.07	0	3.93	-12.95	12.81	0.582
Left eye	Central gaze, y-axis	52	0.01	0	2.76	-9.02	9.04	0.752
Left eye	Upper-left gaze, x-axis	52	-0.16	-1	6.99	-25.74	25.41	0.304
Left eye	Upper-left gaze, y-axis	52	0.19	0	4.71	-18.39	18.77	0.470
Left eye	Upper gaze, x-axis	52	-0.84	0	4.03	-15.66	13.98	0.725
Left eye	Upper gaze, y-axis	52	-0.56	0	3.10	-14.01	12.90	0.579
Left eye	Upper-right gaze, x-axis	52	3.69	0	8.69	-31.02	38.40	0.486
Left eye	Upper-right gaze, y-axis	52	0.54	0	4.63	-14.28	15.36	0.574
Left eye	Left gaze, x-axis	52	-0.71	0	5.40	-20.49	19.07	0.351
Left eye	Left gaze, y-axis	52	0.46	0	2.96	-11.75	12.68	0.661
Left eye	Right gaze, x-axis	52	0.39	0	5.72	-23.94	24.73	0.649
Left eye	Right gaze, y-axis	52	0.74	0	3.30	-13.53	15.01	0.640
Left eye	Lower-left gaze, x-axis	52	-0.66	0	5.07	-18.13	16.80	0.645
Left eye	Lower-left gaze, y-axis	52	0.63	0	3.52	-15.90	17.17	0.354
Left eye	Lower gaze, x-axis	52	-1.06	0	4.50	-14.92	12.80	0.692
Left eye	Lower gaze, y-axis	52	0.13	0	2.10	-7.32	7.59	0.860
Left eye	Lower-right gaze, x-axis	52	-0.78	0	6.78	-27.52	25.96	0.499
Left eye	Lower-right gaze, y-axis	52	-0.12	0	4.50	-16.79	16.56	0.444
Right eye	Central gaze, x-axis	52	1.23	0	3.81	-12.77	15.23	0.450
Right eye	Central gaze, y-axis	52	-1.35	0	3.42	-12.33	9.64	0.654
Right eye	Upper-left gaze, x-axis	52	1.57	0	7.14	-21.98	25.12	0.578
Right eye	Upper-left gaze, y-axis	52	4.33	0	8.10	-24.48	33.13	0.344
Right eye	Upper gaze, x-axis	52	0.20	0	4.68	-17.27	17.67	0.727
Right eye	Upper gaze, y-axis	52	-0.87	0	2.71	-9.59	7.86	0.863
Right eye	Upper-right gaze, x-axis	52	2.09	0	6.24	-22.50	26.67	0.586
Right eye	Upper-right gaze, y-axis	52	2.14	0	4.34	-16.51	20.80	0.647
Right eye	Left gaze, x-axis	52	0.60	0	5.88	-20.17	21.36	0.515
Right eye	Left gaze, y-axis	52	3.04	0	5.15	-16.02	22.09	0.413
Right eye	Right gaze, x-axis	52	-0.84	0	4.22	-15.15	13.48	0.721
Right eye	Right gaze, y-axis	52	0.03	0	2.82	-12.25	12.31	0.766
Right eye	Lower-left gaze, x-axis	52	2.15	0	5.62	-17.74	22.04	0.532
Right eye	Lower-left gaze, y-axis	52	3.33	0	4.85	-14.99	21.64	0.375
Right eye	Lower gaze, x-axis	52	2.51	0	5.32	-21.09	26.11	0.234
Right eye	Lower gaze, y-axis	52	-1.26	0	2.03	-9.48	6.96	0.828
Right eye	Lower-right gaze, x-axis	52	2.13	0	5.81	-17.74	22	0.432
Right eye	Lower-right gaze, y-axis	52	0.86	0	3.99	-15.71	17.42	0.658

Supplementary Table S3. Sensitivity analysis based on participant-eye aggregated measurements.

Eye	n participants	Mean CCC	Mean difference, Δ	95% CI bias, Δ	Mean absolute difference, Δ	Lower LoA, Δ	Upper LoA, Δ
Right eye	52	0.79	1.22	-0.12 to 2.55	3.22	-8.17	10.60
Left eye	52	0.68	0.10	-1.48 to 1.69	3.12	-11.04	11.24

Note. Agreement analysis was repeated after aggregating point-coordinate measurements at the participant-eye level. Abbreviations: CCC, concordance correlation coefficient; CI, confidence interval; LoA, limits of agreement; Δ, prism diopters.

Supplementary Table S4. Full within-method repeatability analysis for point-by-point deviation measurements according to diagnostic gaze position and coordinate component.

Method	Eye	Position/component	n participants	Mean S1, Δ	Mean S2, Δ	Mean difference S2-S1, Δ	Mean absolute difference, Δ	p-value
Conventional Hess-Lancaster	Left eye	Central gaze, x-axis	16	2.50	2.03	-0.47	1.72	1.0000
Conventional Hess-Lancaster	Left eye	Central gaze, y-axis	16	2.66	2.19	-0.47	1.09	1.0000
Conventional Hess-Lancaster	Left eye	Upper-left gaze, x-axis	16	8.28	11.72	3.44	4.06	1.0000
Conventional Hess-Lancaster	Left eye	Upper-left gaze, y-axis	16	3.12	5.47	2.34	3.28	1.0000
Conventional Hess-Lancaster	Left eye	Upper gaze, x-axis	16	6.09	5.47	-0.62	3.44	1.0000
Conventional Hess-Lancaster	Left eye	Upper gaze, y-axis	16	3.44	2.50	-0.94	1.88	1.0000
Conventional Hess-Lancaster	Left eye	Upper-right gaze, x-axis	16	7.66	8.12	0.47	4.84	1.0000
Conventional Hess-Lancaster	Left eye	Upper-right gaze, y-axis	16	5.62	3.91	-1.72	2.66	1.0000
Conventional Hess-Lancaster	Left eye	Left gaze, x-axis	16	4.69	2.66	-2.03	2.34	1.0000
Conventional Hess-Lancaster	Left eye	Left gaze, y-axis	16	2.81	4.22	1.41	2.03	1.0000
Conventional Hess-Lancaster	Left eye	Right gaze, x-axis	16	7.50	7.97	0.47	2.03	1.0000
Conventional Hess-Lancaster	Left eye	Right gaze, y-axis	16	3.59	4.22	0.62	1.88	1.0000
Conventional Hess-Lancaster	Left eye	Lower-left gaze, x-axis	16	4.22	1.72	-2.50	4.69	1.0000
Conventional Hess-Lancaster	Left eye	Lower-left gaze, y-axis	16	2.50	1.56	-0.94	1.88	1.0000
Conventional Hess-Lancaster	Left eye	Lower gaze, x-axis	16	3.44	0.78	-2.66	3.59	1.0000
Conventional Hess-Lancaster	Left eye	Lower gaze, y-axis	16	1.56	0.62	-0.94	1.25	1.0000
Conventional Hess-Lancaster	Left eye	Lower-right gaze, x-axis	16	7.81	4.22	-3.59	4.22	1.0000
Conventional Hess-Lancaster	Left eye	Lower-right gaze, y-axis	16	5	2.19	-2.81	5	1.0000
Conventional Hess-Lancaster	Right eye	Central gaze, x-axis	16	4.38	3.44	-0.94	2.19	0.6948
Conventional Hess-Lancaster	Right eye	Central gaze, y-axis	16	3.75	3.28	-0.47	0.78	0.6948
Conventional Hess-Lancaster	Right eye	Upper-left gaze, x-axis	16	7.03	7.81	0.78	6.09	0.6948
Conventional Hess-	Right eye	Upper-left gaze, y-axis	16	2.50	2.97	0.47	1.72	0.6948

Method	Eye	Position/component	n participants	Mean S1, Δ	Mean S2, Δ	Mean difference S2-S1, Δ	Mean absolute difference, Δ	p-value
Lancaster								
Conventional Hess-Lancaster	Right eye	Upper gaze, x-axis	16	5.47	6.41	0.94	1.88	0.6948
Conventional Hess-Lancaster	Right eye	Upper gaze, y-axis	16	2.97	3.28	0.31	0.62	0.6948
Conventional Hess-Lancaster	Right eye	Upper-right gaze, x-axis	16	7.50	7.66	0.16	2.97	0.6948
Conventional Hess-Lancaster	Right eye	Upper-right gaze, y-axis	16	4.53	5.16	0.62	1.88	0.6948
Conventional Hess-Lancaster	Right eye	Left gaze, x-axis	16	5.62	3.75	-1.88	3.44	0.6948
Conventional Hess-Lancaster	Right eye	Left gaze, y-axis	16	2.50	3.75	1.25	2.19	0.6948
Conventional Hess-Lancaster	Right eye	Right gaze, x-axis	16	6.88	3.12	-3.75	4.06	0.6948
Conventional Hess-Lancaster	Right eye	Right gaze, y-axis	16	5.16	4.69	-0.47	1.72	0.6948
Conventional Hess-Lancaster	Right eye	Lower-left gaze, x-axis	16	3.44	4.06	0.62	3.75	0.6948
Conventional Hess-Lancaster	Right eye	Lower-left gaze, y-axis	16	1.88	2.19	0.31	2.50	0.6948
Conventional Hess-Lancaster	Right eye	Lower gaze, x-axis	16	3.12	2.50	-0.62	4.38	0.6948
Conventional Hess-Lancaster	Right eye	Lower gaze, y-axis	16	4.53	2.50	-2.03	3.91	0.6948
Conventional Hess-Lancaster	Right eye	Lower-right gaze, x-axis	16	7.34	4.06	-3.28	4.84	0.6948
Conventional Hess-Lancaster	Right eye	Lower-right gaze, y-axis	16	4.22	5.16	0.94	2.19	0.6948
Dicopt Pro	Left eye	Central gaze, x-axis	14	4.57	4.36	-0.21	0.64	0.5751
Dicopt Pro	Left eye	Central gaze, y-axis	14	3.43	3.93	0.50	0.64	0.5751
Dicopt Pro	Left eye	Upper-left gaze, x-axis	14	9	9.07	0.07	3.79	0.5751
Dicopt Pro	Left eye	Upper-left gaze, y-axis	14	5.71	8.36	2.64	2.79	0.5751
Dicopt Pro	Left eye	Upper gaze, x-axis	14	6.29	6.43	0.14	1.43	0.5751
Dicopt Pro	Left eye	Upper gaze, y-axis	14	5.14	3.79	-1.36	3.07	0.5751
Dicopt Pro	Left eye	Upper-right gaze, x-axis	14	12.57	13.21	0.64	2.64	0.5751
Dicopt Pro	Left eye	Upper-right gaze, y-axis	14	8.57	10.86	2.29	3.29	0.5751
Dicopt Pro	Left eye	Left gaze, x-axis	14	4.43	4.21	-0.21	1.50	0.5751
Dicopt Pro	Left eye	Left gaze, y-axis	14	4.71	3.36	-1.36	1.64	0.5751
Dicopt Pro	Left eye	Right gaze, x-axis	14	8.14	7.43	-0.71	3	0.5751
Dicopt Pro	Left eye	Right gaze, y-axis	14	6.79	5.36	-1.43	2.71	0.5751
Dicopt Pro	Left eye	Lower-left gaze, x-axis	14	5.14	4.86	-0.29	1.14	0.5751
Dicopt Pro	Left eye	Lower-left gaze, y-axis	14	4.79	4.79	0	0.86	0.5751
Dicopt Pro	Left eye	Lower gaze, x-axis	14	5.29	5.86	0.57	1.29	0.5751
Dicopt Pro	Left eye	Lower gaze, y-axis	14	3.21	3.86	0.64	0.79	0.5751

Method	Eye	Position/component	n participants	Mean S1, Δ	Mean S2, Δ	Mean difference S2-S1, Δ	Mean absolute difference, Δ	p-value
Dicopt Pro	Left eye	Lower-right gaze, x-axis	14	7.93	9.79	1.86	3.43	0.5751
Dicopt Pro	Left eye	Lower-right gaze, y-axis	14	4.93	4.14	-0.79	3.07	0.5751
Dicopt Pro	Right eye	Central gaze, x-axis	14	5	4.93	-0.07	2.50	0.0159
Dicopt Pro	Right eye	Central gaze, y-axis	14	4.29	4.64	0.36	1.07	0.0159
Dicopt Pro	Right eye	Upper-left gaze, x-axis	14	3.93	9.86	5.93	7.36	0.0159
Dicopt Pro	Right eye	Upper-left gaze, y-axis	14	5.36	18.14	12.79	13.50	0.0159
Dicopt Pro	Right eye	Upper gaze, x-axis	14	6.57	7.57	1	3.43	0.0159
Dicopt Pro	Right eye	Upper gaze, y-axis	14	4.57	5.07	0.50	1.36	0.0159
Dicopt Pro	Right eye	Upper-right gaze, x-axis	14	7	9.86	2.86	5.29	0.0159
Dicopt Pro	Right eye	Upper-right gaze, y-axis	14	6.07	7.79	1.71	3.14	0.0159
Dicopt Pro	Right eye	Left gaze, x-axis	14	5.21	6.07	0.86	1.86	0.0159
Dicopt Pro	Right eye	Left gaze, y-axis	14	6.36	4.64	-1.71	2.43	0.0159
Dicopt Pro	Right eye	Right gaze, x-axis	14	4.29	5.21	0.93	2.21	0.0159
Dicopt Pro	Right eye	Right gaze, y-axis	14	4.43	4.93	0.50	1.07	0.0159
Dicopt Pro	Right eye	Lower-left gaze, x-axis	14	5.79	8.50	2.71	2.71	0.0159
Dicopt Pro	Right eye	Lower-left gaze, y-axis	14	3.21	4.29	1.07	2.07	0.0159
Dicopt Pro	Right eye	Lower gaze, x-axis	14	4.50	5.57	1.07	1.64	0.0159
Dicopt Pro	Right eye	Lower gaze, y-axis	14	3.21	2.71	-0.50	0.93	0.0159
Dicopt Pro	Right eye	Lower-right gaze, x-axis	14	4.07	7.50	3.43	3.71	0.0159
Dicopt Pro	Right eye	Lower-right gaze, y-axis	14	4.36	7.29	2.93	4.21	0.0159
